# Prevalence of White Coat Hypertension Among Surgical Patients at a Tertiary Care Hospital: A Cross-Sectional Observational Study

**DOI:** 10.7759/cureus.89308

**Published:** 2025-08-03

**Authors:** Aiman Shah, Azhar Rehman

**Affiliations:** 1 Department of Anesthesiology, Aga Khan University Hospital, Karachi, PAK

**Keywords:** blood pressure, prevalence, surgical patients, tertiary care hospital, white coat hypertension

## Abstract

Background

The prevalence of white coat hypertension (WCH) among surgical patients is a significant concern. WCH refers to a condition where individuals with normal blood pressure at home exhibit elevated readings when measured at a medical facility, affecting clinical decision-making processes. The aim is to determine the prevalence of WCH among patients undergoing elective surgery at a tertiary care hospital and to evaluate its association with demographic factors, surgical type, and perioperative waiting time. A cross-sectional design was employed, enrolling 150 patients. The findings reveal a significant prevalence of WCH, especially among female patients, with varying prevalence rates across different surgical procedures. These results underscore the importance of recognizing WCH to avoid unnecessary delays or cancellations of surgeries and to implement appropriate management strategies that can enhance patient outcomes.

Methodology

This cross-sectional study assesses the prevalence of WCH among surgical patients at a tertiary care hospital in Karachi, Pakistan. The study was carried out at the preoperative assessment clinic, Aga Khan University Hospital, Karachi, from April 1, 2024, to September 30, 2024. One hundred and fifty patients were enrolled. All patients aged 18 to 75 years, of either gender, scheduled to undergo elective surgery were included. Blood pressure was measured as a routine by the preoperative assessment clinic staff using a standard mercury sphygmomanometer using accurate cuff sizes. Patients were instructed to monitor their ambulatory blood pressure at home. They were asked to maintain a detailed blood pressure chart, recording the date, time, and blood pressure values. This chart was reviewed by the research team on the day of surgery and used for comparison with clinic readings to assess for WCH.

Results

The prevalence of WCH was 41.3% (n=62). Female (n=43) patients comprised a greater proportion (70%) of the total WCH cases. Around 33.3% patients were only those who presented a previous history of hospital admission, while 66.7% were those having primary admission. The most prevalent WCH was found in tumor excision cases (53.6%), followed by the renal surgery group (45.2%), then neurosurgery cases (42.5%). It was observed that there was an increased blood pressure range of ambulant daytime systolic and diastolic values in WCH cases in comparison to nighttime values. There was a significantly higher number of cases that had to wait longer before surgery in the preoperative holding area (p=0.002).

Conclusion

The prevalence of WCH is significant among surgical patients. Early diagnosis and prompt management could reduce complications and the cancellation rate as well. All these findings emphasized the need to differentiate between essential hypertension and WCH, and once it is identified, it should be treated for a better outcome and to avoid case cancellation. Additionally, WCH has been associated with a greater risk of cardiovascular mortality compared to prehypertension. Recommendations support the use of non-pharmacological interventions for mild to moderate white coat hypertension (WCH), while pharmacological treatment may be considered in more severe cases. This approach differs from the management of essential hypertension.

## Introduction

Hypertension has become increasingly prevalent in recent times, affecting over a billion individuals globally. According to the World Hypertension Society and the International Society of Hypertension, approximately 60% of the adult population worldwide meets the criteria for hypertension [1]. Many patients undergoing surgery may have uncontrolled or insufficiently managed hypertension, significantly elevating their perioperative risk for complications such as myocardial infarction, ischemia, stroke, arrhythmias, and renal failure [2-5].

A distinct condition known as white coat hypertension (WCH) occurs when individuals who typically have normal blood pressure experience elevated readings upon entering a medical facility. This phenomenon creates a clinical challenge in determining whether to proceed with the scheduled surgery or postpone it [6]. The global prevalence of WCH ranges between 15% and 30% [7]. It is more commonly observed in women, older adults, non-smokers, pregnant individuals, and those without signs of target organ damage. Research indicates that individuals with WCH have a higher likelihood of developing sustained hypertension over a span of five to ten years [8].

The primary cause of WCH is often an exaggerated response to anxiety. Even minor surgical procedures, such as dental treatments, can trigger significant anxiety due to factors like fear of pain and concern about the surgical outcome. Managing preoperative anxiety is a crucial aspect of anesthetic practice. Non-pharmacological approaches include psychotherapy, acupressure, and music therapy, while pharmacological options involve medications such as benzodiazepines (diazepam, midazolam), alpha-2 agonists (clonidine), gabapentin, and melatonin [9,10].

This study aimed to determine the prevalence of WCH and explore its associations with demographic and surgical factors among elective surgery patients. The patients suffering from WCH were identified. In the present study, a detailed analysis of patients for WCH was made before any surgical procedure. The patients suffering from WCH were identified. The results of this study have led to the formulation of substantial data that assist in controlling anxiety in patients against WCH and ease in surgical process. The present study provides evidence-based results for minimizing hypertension among patients and provides positive health outcomes.

The aim is to determine the prevalence of WCH among patients undergoing elective surgery at a tertiary care hospital and to evaluate its association with demographic factors, surgical type, and perioperative waiting time. The following are the objectives: (i) To identify demographic and clinical factors associated with WCH in surgical patients; (ii) To compare ambulatory and clinic blood pressure readings to confirm WCH; (iii) To assess the impact of WCH on surgical workflow, including delays and waiting time in the preoperative area; (iv) To highlight the need for distinguishing WCH from essential hypertension for appropriate perioperative management.

## Materials and methods

This cross-sectional study was conducted at the preoperative assessment clinic, Aga Khan University Hospital, Karachi, from April 1, 2024, to September 30, 2024. The Ethics Review Committee, Aga Khan University, Karachi, issued approval number 2024-9148-28263. A total of 150 patients taking frequency of preoperative hypertension to be 54%, a margin of error of 8% and a confidence level (CI) of 95% were enrolled. This sample size was calculated using Epi Info, version 7 (Centers for Disease Control and Prevention, Atlanta, GA, USA). Informed consent was gained from each patient enrolled before initiation of research. The informed consent had the total details of risks, benefits, and federal rights of the patient associated with participation in the research.

All patients scheduled to undergo elective surgery, either gender and age 18 years and up to 75 years, were included. The patients with ADHD (attention-deficit/hyperactivity disorder), history of mania, bipolar effective disorder or post-traumatic stress, history of diabetes mellitus, hypertension, metabolic syndromes, hypothyroidism or hyperthyroidism, obstetric patients, and history of substance abuse or self-medication history, were excluded.

A history of demographic information was taken from the patients. The method of the study was based on one-to-one interviewing, ensuring the confidentiality of the patients as a priority. The blood pressure was measured as a routine by the pre-operative assessment clinic staff using a standard mercury sphygmomanometer with an accurate cuff size. In addition to clinic blood pressure measurements, patients were instructed to monitor their ambulatory blood pressure at home. They were asked to maintain a detailed blood pressure chart, recording the date, time, and systolic and diastolic values. This chart was reviewed by the research team on the day of surgery and used for comparison with clinic readings to assess for white coat hypertension. 

The reporting time of all patients, waiting time before surgery, and type of surgery were included in the collected data. A well-structured questionnaire was designed to document all the relevant information of the study (see Appendices). Each patient’s detailed educational, marital status, occupational, and smoking history was analyzed in addition to the clinical history. The weight, height, and BMI measurements, as well as blood pressures, were measured. The surgical procedures were sub-typed and their association with the WCH was assessed.

WCH was estimated as the prevalence among the total enrolled patients. It was interpreted as elevated office BP (≥140 and/or 90 mmHg). Previously non-hypertensive patients who had blood pressure >140/90 mm Hg were labeled as white coat hypertension. A comparison of WCH patients was done with normotensives in terms of their BP values within day and night for a detailed assessment of blood pressure changes associated with WCH.

Data was analyzed on IBM SPSS Statistics for Windows, version 26 (IBM Corp., Armonk, NY, USA), wherein chi-square and odds ratio were applied for analyses. P value <0.001 was considered significant.

Patients used validated digital blood pressure (BP) monitors at home. They were instructed to take two readings (morning and evening) for five days while seated, after five minutes of rest, and to record values in a provided BP diary. In case high BP was observed, it was measured once again after 10 minutes of rest. Those patients with little elevation with no symptoms were observed without treatment. In case of high and dangerous levels of BP (e.g., >180 / 110 mmHg) or the presence of symptoms, short-acting antihypertensives were applied with the advice of the anesthesiology team.

A validated digital BP monitor (Omron HEM-7121, Omron Corporation, Kyoto, Japan) was used to train patients in a standardized way. They also calculated BP twice a day at 5 days, sitting in rested poses, and took it through a well-organized logbook. WCH was characterized by clinic BP is 140/90 mmHg (average of two readings taken two minutes apart after a five-minute rest) or more, and home BP is less than 135/85 mmHg (average of all valid readings recorded over five days). The diagnosis was made with reference to average readings, with American Heart Association (AHA)/European Society of Hypertension (ESH) recommendations in the case of lack of ambulatory blood pressure monitoring (ABPM) [11].

Waiting time was retrieved from electronic hospital records. It was measured based on the time spent in the pre-op holding area before being transferred (see Appendices). The study admits the issue of possible measurement bias. The blood pressure in all the clinics was taken by qualified nurses, and these measurements involved the use of appropriately sized cuffs and mercury sphygmomanometers. All of the measurements were done when the patient was seated, five minutes after rest, and with the arm in a heart-level position. Each session would give two readings with two-minute intervals, and the average was taken. This real-world preoperative state did not allow blinding procedures, and to reduce the inter-observer variability, measurements of all the parameters were conducted with a standardized protocol. It was also important to make the environmental conditions as constant as possible.

## Results

This study enrolled a total of 150 adult patients, aged between 18-75 years; 57.33% were female patients. The mean age of the patients was 44.5±4.4 years, with the majority coming from a rural background, most of them having at least a matriculation level of education. Unemployment was observed in 29.3% while the mean value of weight was calculated as 27.1±1.6 kg/m2. Mean systolic BP of the patients in the perioperative period was observed as 153.5±8.5 mmHg and diastolic 96.4±6.7 mmHg (Tables 1, 2).

**Table 1:** Detailed demographics of the patients (n=150)

Variable	Frequency	Percentage	Test statistics (Chi-square)	P value
Gender				
Male	64	42.6	8.45	<0.001
Female	86	57.4		
Resident				
Rural	77	51.3	7.57	0.006
Urban	73	48.6		
Marital status				
Married	78	52.0	7.98	0.005
Unmarried	72	48.0		
Educational status				
< Matriculation	30	20.0	4.75	0.033
> Matriculation	120	80.0		
Employment status				
Employed	61	40.6	10.32	<0.001
Unemployed	44	29.3		
Home makers	45	30.0		
Family history of systolic hypertension				
Yes	102	68.0	8.72	0.004
No	48	32.0		
Smoking				
Yes	48	32.0	10.47	<0.001
No	102	68.0		

**Table 2:** Frequency of white coat hypertension (WCH) against different variables (n=62)

Variable	Category	WCH (n, %)	Test statistics (Chi-square)	P-value
Gender	Male (n=64)	19 (29.7%)	3.85	0.050
	Female (n=86)	43 (50.0%)		
Age (years)	35–45 (n=100)	42 (42.0%)	4.20	0.040
	46–56 (n-50)	20 (40.0%)		
Education	< Matriculation (n=30)	14 (46.7%)	5.10	0.025
	> Matriculation (n=120)	48 (40.0%)		
Residence	Rural (n=77)	32 (41.6%)	2.65	0.103
	Urban (n=73)	30 (41.1%)		
Marital status	Married (n=78)	32 (41.0%)	1.80	0.181
	Unmarried (n=72)	30 (41.7%)		
Employment status	Employed (n=61)	22 (36.1%)	6.28	0.012
	Unemployed (n=44)	20 (45.5%)		
	Home makers (n=45)	20 (44.4%)		
Family history of hypertension	Yes (n=102)	45 (44.1%)	3.40	0.065
	No (n=48)	17 (35.4%)		
Smoking	Yes (n=48)	22 (45.8%)	5.40	0.020
	No (n=102)	40 (39.2%)		

Overall, WCH was found in 62 (41.3%) patients. Around 32.3% patients were those who had a previous history of hospital admission, while 67.7% were those having primary admission. The most prevalent WCH was found in tumor excision cases (53.6%), followed by renal surgery (45.2%) and neurosurgery (42.5%), respectively.

The ambulatory hemodynamic parameters of the participants with WCH and normal blood pressure were assessed. It was observed that there was an increased BP range of ambulant day time systolic and diastolic values in WCH cases in comparison to night time values. The similar scenario was observed in normotensive cases as well with significant variations (Table 3).

**Table 3:** Ambulatory hemodynamic parameters of the patients with WCH and normal blood pressure WCH: white coat hypertension; SBP: systolic blood pressure; DBP: diastolic blood pressure

Variables	WCH	Normotensive	Test statistics	ρ-value
Ambulant daytime SBP	124.4±2.8	117.7±3.6	t=3.48	< 0.001
Ambulant daytime DBP	74.8±2.9	69.8±4.0	t= 3.92	< 0.001
Ambulant nighttime SBP	118.3±7.2	108.7±7.7	t=4.15	< 0.001
Ambulant nighttime DBP	70.4±6.2	61.9±6.1	t=4.29	< 0.001

There was significantly longer waiting time before moving the patients from pre operative holding area into the operating room (p=0.002). Only 8% of the patients waited for one hour in preoperative holding area, 11% waited for one to two hours, and 22% of the patients waited for more than two hours before being wheeled into the operating room.

## Discussion

This study aimed to assess the prevalence of WCH among patients undergoing elective surgery. The white coat phenomenon is relatively common in clinical settings. Our findings revealed that 41.3% of participants coming for elective surgery had WCH, which is a huge number. The WCH prevalence in our study is a little bit higher than the available data, which shows a prevalence of WCH around 15-30% [12-14]. This could be due to the difference in education level as the literature has shown a direct relationship of WCH and education level [15], or it could be related to other factors which predispose patient to anxiety and causing WCH or higher observed prevalence may reflect differences in measurement methodology, particularly the reliance on self-reported home BP data rather than ABPM. Accurately identifying these conditions is essential to prevent unnecessary cancellation of elective cases and use of long-term antihypertensive treatment and to ensure appropriate follow-up care. This study shows that patients younger than 46 years and female individuals have more WCH, which is contrary to the current available literature, which found WCH to be more prevalent in older patients [16]. However, it is well established that younger individuals tend to experience higher levels of anxiety, which may contribute to the development of WCH.

This study does not show any strong correlation between WCH and marital status. Similarly, the area of residence and employment status do not have any impact on its prevalence. In our study, smoking also does not contribute to WCH, although previous studies have shown that the occurrence of WCH is more in nonsmokers [17]. Our study shows that the type of surgery has a significant impact on WCH. Patients undergoing tumor resection surgery exhibited a higher prevalence of WCH, which is understandable given their typically prolonged treatment course (including chemotherapy or radiotherapy) that may heighten anxiety and contribute to the development of WCH.

This study shows that WCH is associated with increased waiting time before surgery in the preoperative holding area, presenting an increasing level of anxiety among patients. Hartle et al. reported that preoperative blood pressure is negatively influenced by an increase in waiting time [18]. Another finding in this study is that ambulatory parameters of the participants were in the increased BP range of ambulant daytime in WCH cases in comparison to nighttime values. In comparison, Patterson et al. reported that suspected WCH accounted for 10.6% of ABPM referrals [19]. However, other studies focused on the role of ABPM in hypertension risk assessment and management rather than determining the exact prevalence of WCH [20-23].

All these findings emphasized the need to differentiate between essential hypertension and WCH, and once identified, to be treated for better patient outcomes and to avoid case cancellation. Additionally, WCH has been associated with a greater risk of cardiovascular mortality compared to prehypertension [12-14].

Limitations

The out-of-clinic BP information was not carefully measured due to patient self-reporting, and the ambulatory BP information was not carefully standardized in time and pre-test conditions (i.e., post-waking and caffeine). Although the initiative to teach proper technique was employed, and patients were recommended not to do something that might have interfered with the process, compliance could not be proved objectively. This creates some form of variability and reduces the ability to exclude vital hypertension, which is one of the study design limitations.

There are a number of limitations of this study that need to be noted in consideration of the conclusions of the paper. First, the diagnosis of WCH was performed using self-reported home BP diaries as opposed to standardized 24-hour ABPM, which is regarded as the gold standard diagnostic measure. Lack of ABPM can interfere with the accuracy of diagnosis and create a possibility of classification bias. Patients had undergone training on appropriate BP measurement technique, but compliance with instructions in terms of timing (e.g., after waking), rest conditions, and the exclusion of confounding effects (the consumption of caffeine or recent exercise) could not be checked independently.

Second, there might have been measurement bias because the diagnosis of blood pressure in the clinic was not standardized. The level of readings, time of rest, consistency of the observer, and control of the environment were not carefully documented and monitored, which enhances the chances of inter-observer and temporal differences. This could have affected the accuracy of BP readings collected in the office in establishing high BP.

Third, it has low external validity of its results. Since the research team studied a sample of patients in only one tertiary care facility in Karachi, and the population of patients includes a high percentage of rural dwellers and low educational levels, one may not be able to apply the study results to another setting or population.

Fourth, being a cross-sectional study, our design imposes this limitation on the detection of causality. Although there were some reported associations between WCH, preoperative waiting time, gender, and type of surgery, these associations could not be anticipated as cause-and-effect conclusions.

Fifth, although in its list of potential variables contributing to a high preoperative BP, anxiety is discussed, no validated psychometric assessment of anxiety levels or its possible role as a mediating variable was used. This restrains the theoretical force of our argument between WCH and psychological stress. It was hypothesized that such confounders as level of education and surgical anxiety could be at the basis of the seemingly high prevalence rates of WCH in our group, but that was not proved by the statistical analysis. Future studies would have to be more rigorous in testing these hypotheses.

Recommendations

Nonetheless, the study also raises a significant yet little-discussed topic in the area of perioperative care. Future studies are, however, recommended to include standardized diagnostic equipment (ABPM), standardized anxiety scales, and multi-center designs to enhance the accuracy, repeatability, and applicability. As a result, the potential for misclassification and measurement variability should be considered when interpreting the prevalence and associations reported in this study.

The American Heart Association and the American College of Cardiology recommend non-pharmacological methods for mild to moderate WCH and pharmacological therapy for severe WCH, which is different from essential hypertension [24]. The incorporation of ambulatory blood pressure measurement or an overseen home BP monitoring should be introduced in future studies to provide better diagnostic accuracy and deal with possible biases of the measures.

## Conclusions

The prevalence of WCH is significantly higher among surgical patients. Drawing conclusions, it can be summarized that WCH is very common among surgical patients and possibly causes unnecessary treatment or delays in an operation in the instance that it is not detected properly. This study fulfilled its aim by estimating the prevalence of WCH among elective surgical patients and identifying associated factors, without drawing causal or interventional conclusions. It is crucial to underline the importance of adequate BP assessment measures, and utilization of home BP measurement or ABPM in cases of their availability, as the way to distinguish between WCH and essential hypertension and provide an adequate perioperative process.

## Appendices

Structured questionnaire

Section A: Demographics

1.     Name: ___________________________

2.     Age: _______

3.     Gender: ☐ Male ☐ Female

4.     Marital Status: ☐ Married ☐ Unmarried

5.     Residential Area: ☐ Urban ☐ Rural

6.     Educational Level:

☐ No formal education ☐ Below matriculation ☐ Matriculation ☐ Intermediate ☐ Graduate or above

7.     Employment Status:

☐ Employed ☐ Unemployed ☐ Homemaker ☐ Retired

Section B: Medical and Surgical History

1.     Previous hospital admissions: ☐ Yes ☐ No

o   If yes, reason: ______________________

2.     Previous diagnosis of hypertension: ☐ Yes ☐ No

3.     Family history of hypertension: ☐ Yes ☐ No

4.     Known medical conditions (select all that apply):

☐ Diabetes ☐ Thyroid disorder ☐ Cardiac disease ☐ Psychiatric illness ☐ None

5.     Current medications (list): ______________________

Section C: Lifestyle Factors

1.     Do you smoke? ☐ Yes ☐ No

o   If yes: ☐ Occasionally ☐ Regularly

2.     Alcohol consumption: ☐ Yes ☐ No

3.     Physical activity (days per week): ______

4.     Self-medication history: ☐ Yes ☐ No

Section D: Preoperative Information

1.     Type of surgery scheduled: ______________________

2.     Date of surgery: ______________

3.     Reporting time to hospital: _______ AM/PM

4.     Time wheeled into OR: _______ AM/PM

5.     Estimated waiting time in pre-op holding area: ______ minutes

6.     Any anxiety symptoms before surgery? ☐ Yes ☐ No

o   If yes, rate your anxiety on a scale of 0-10: ______

Section E: Blood Pressure Monitoring Log (Patient-Reported)

**Table 4:** Blood pressure monitoring log

Date	Time	Systolic (mmHg)	Diastolic (mmHg)	Notes (e.g., posture, symptoms)
-	-	-	-	-
